# Pressure Adaptations in Deep-Sea *Moritella* Dihydrofolate Reductases: Compressibility versus Stability

**DOI:** 10.3390/biology10111211

**Published:** 2021-11-20

**Authors:** Ryan W. Penhallurick, Toshiko Ichiye

**Affiliations:** Department of Chemistry, Georgetown University, Washington, DC 20057, USA; rwp33@georgetown.edu

**Keywords:** deep-sea adaptations, compressibility, cavities, pressure, potential energy landscape

## Abstract

**Simple Summary:**

Deep-sea organisms must have proteins that function under high hydrostatic pressure to survive. Adaptations used in proteins from “pressure-loving” piezophiles may include greater compressibility or greater stability against pressure-induced destabilization. However, while greater compressibility can be accomplished by greater void volume, larger cavities in a protein have been associated with greater destabilization and even unfolding as pressure is increased. Here, computer simulations of dihydrofolate reductase from a moderate piezophile and a hyperpiezophile were performed to understand the balance between adaptations for greater compressibility and those against pressure destabilization and unfolding. The results indicate that while compressibility appears to be important for deep-sea microbes, adaptation for the greatest depths may be to prevent water penetration into the interior.

**Abstract:**

Proteins from “pressure-loving” piezophiles appear to adapt by greater compressibility via larger total cavity volume. However, larger cavities in proteins have been associated with lower unfolding pressures. Here, dihydrofolate reductase (DHFR) from a moderate piezophile *Moritella profunda* (Mp) isolated at ~2.9 km in depth and from a hyperpiezophile *Moritella yayanosii* (My) isolated at ~11 km in depth were compared using molecular dynamics simulations. Although previous simulations indicate that MpDHFR is more compressible than a mesophile DHFR, here the average properties and a quasiharmonic analysis indicate that MpDHFR and MyDHFR have similar compressibilities. A cavity analysis also indicates that the three unique mutations in MyDHFR are near cavities, although the cavities are generally similar in size in both. However, while a cleft overlaps an internal cavity, thus forming a pathway from the surface to the interior in MpDHFR, the unique residue Tyr103 found in MyDHFR forms a hydrogen bond with Leu78, and the sidechain separates the cleft from the cavity. Thus, while *Moritella* DHFR may generally be well suited to high-pressure environments because of their greater compressibility, adaptation for greater depths may be to prevent water entry into the interior cavities.

## 1. Introduction

The discovery of life thriving under extreme conditions of temperature, pressure, and composition has led to intriguing questions about the limits at which life can survive. Mechanisms used by “extremophiles” to adapt their biological macromolecules to these extremes could assist in our understanding of these limits of life. Studying the sequence-structure-function relationship of proteins from extremophiles compared to proteins from organisms living under ambient conditions, “mesophiles,” is useful in understanding adaptations used to maintain functional enzymes under all conditions [1]. So far, studies of extremophiles have largely focused on temperature adaptation. For instance, the corresponding states hypothesis that enzyme activity is high near the growth conditions (i.e., growth temperature *T*_G_ and growth pressure *P*_G_) of the microbe [2] and that maximal activity is achieved by balancing the stability and flexibility of the protein [3] is mainly based on studies of homologous enzymes of temperature-adapted microbes. However, less is understood about adaptations to high hydrostatic pressure largely due to limited access to extreme oceanic depths until recently. Since about 88% of the ocean has biologically high pressures, comprising the largest portion of the biosphere [4,5], and “piezophiles” have now been found in a wide range of environments, including hydrothermal vents, deep-sea trenches, and under the Earth’s crust [6,7], studies of adaptation to high pressure are timely.

For proteins, the effects of pressure are compression and denaturation [8]. Below 4 kbar, proteins mostly compress, while generally far above ~4 kbar, single-chain proteins will denature. Although seemingly contrary to volume reduction necessary to lower the free energy, pressure unfolding apparently occurs since water can pack more tightly against polypeptide than polypeptide against polypeptide so that more open solvated states become favorable [9], thus lowering the free energy of the entire system.

Numerous studies of pressure unfolding of proteins have shown that larger cavity size within a protein apparently leads to lower pressure stability [10] because the system volume is lowered when water enters these cavities. Point mutants that increase the cavity volume decrease the stability of the enzyme as pressure increases [11], and even slight, local changes affecting cavity sizes can have profound impacts on destabilization, as well as on the conformational dynamics [12]. Moreover, pressure-induced water penetration into internal cavities of proteins is observed in high-pressure crystallography [13,14] and high-pressure NMR [15].

However, adaptations for smaller cavity volumes in piezophile proteins have not been found. In fact, comparisons of crystal structures of 3-isopropyl malate dehydrogenase (IPMDH) from the obligate piezophile *Shewanella benthica* (Sb) with that from the mesophile *Shewanella oneidensis* (So) find a larger total cavity volume in SbIPMDH, although this was attributed to more numerous small cavities rather than larger cavities [16]. Furthermore, the larger total cavity volume in SbIPMDH was proposed as an adaptation for greater compressibility. In addition, although experimental determination of the compressibilities of proteins is complicated by the need to measure the proteins in solution, studies of the effects of ligand binding [17] and single-point mutations [18] find that relatively small changes to local structure can have large effects on both the compressibility and activity of an enzyme.

Dihydrofolate reductase (DHFR) is an ~160 residue, ~20 kDa enzyme that is a prime target for comparative studies of extremophile proteins because it is ubiquitous. DHFR catalyzes the hydride transfer and protonation of dihydrofolate (DHF) from the coenzyme NADPH to form tetrahydrofolate (THF), an essential precursor in the purine biosynthesis pathway [19,20]. Conformational changes of the loops and subdomains have been found to be important in the catalytic cycle [21,22,23,24]. In addition, extensive experimental studies have been compared for DHFR from the mesophile (*T*_G_ = 37 °C, *P*_G_ = 1 bar) *Escherichia coli* (Ec) with that from the moderate psychropiezophile (*T*_G_ = 6 °C, *P*_G_ = 220 bar [25]) *Moritella profunda* (Mp) [26,27,28], as well as other deep-sea piezophiles [29,30,31]. Although major structural differences are not apparent between crystal structures of MpDHFR and those of EcDHFR [27], MpDHFR has maximum enzyme activity at 500 bar while EcDHFR is monotonically inactivated by pressure above 1 bar. Moreover, MpDHFR appears to have a larger total cavity volume than EcDHFR [27], so it appears to be adapted by having greater compressibility. Another potential adaptation in MpDHFR is the presence of Glu27 rather than Asp27 in EcDHFR. With increasing pressure, the Asp27Glu mutation (D27E) of EcDHFR exhibits increased activity rather than the decreased activity observed in wild-type EcDHFR [28]. However, while Glu27 has been found in all species of *Moritella* DHFR so far, it is not in other piezophile DHFR, indicating it may allow but is not necessary for high-pressure activity [32]. In addition, enzyme activity does not always increase with pressure for DHFR from other deep-sea bacteria from other genera [33]. In addition, the unfolding pressure (*P*_u_) at 25 °C is 2.7 kbar for apo-EcDHFR but only 0.7 kbar for apo-MpDHFR, indicating MpDHFR is actually more sensitive to pressure denaturation than EcDHFR [27]. The marginal stability of DHFR and other enzymes from other deep-sea piezophiles has been noted [33]. Since many of the piezophile proteins studied have been from the cold deep ocean, the marginal stability may be an adaptation for cold temperature rather than for high pressure [34].

Molecular dynamics (MD) simulation can provide an important molecular perspective to experimental studies. Our previous MD simulation studies of EcDHFR and MpDHFR showed that MpDHFR had higher overall atomic fluctuations than EcDHFR, and pressure appeared to increase fluctuations [35,36]. The higher fluctuations in MpDHFR appeared to be due to somewhat fewer hydrogen bonds in MpDHFR compared to EcDHFR. Comparisons of MpDHFR versus EcDHFR [37] and of wild-type versus D27E EcDHFR [38] indicate that strengthening of the strong Thr113 O…Asp27 O*_δ_* hydrogen bond under pressure leads to the monotonic pressure deactivation in EcDHFR by overcorrelating collective motions while strengthening of the weak Thr113 O…Glu27 O*_ε_* hydrogen bond to a strength similar to EcDHFR at 1 bar leads the pressure activation in MpDHFR and D27E EcDHFR.

In addition, since the compressibility of a protein is difficult to measure, a quasiharmonic analysis (QHA) is a method based on computer simulations that allows another assessment of compressibility. A QHA probes the local energy landscape through a series of short simulations at a variety of temperatures and pressures around a reference set of conditions [39]. The effective local potential well for a given atom created by its neighbors is assumed to be described by the atomic fluctuations, and the temperature and pressure dependence are defined at a set of reference conditions by the width of the average well *σ*_0_^2^, an intrinsic isobaric expansivity *α*_P_*,* and an intrinsic isothermal compressibility *κ*_T_. At 279 K, 1 bar, the QHA indicates MpDHFR (*κ*_T_ = 76 × 10^−3^ / kbar) was more compressible compared to EcDHFR (*κ*_T_ = 67 × 10^−3^/kbar) [40], consistent with the crystallographic studies.

Finally, our previous sequence comparison and molecular dynamics studies of DHFR from *Moritella* [41] indicate that in general, *Moritella* DHFR may have been adapted for the cold by having fewer interactions so that they are more flexible, but that this adaptation may also be fortuitously favorable for high pressures by making them more compressible. However, weaker interactions also would lead to lower stability under either or both higher temperatures or higher pressures. DHFR from *Moritella yayanosii* (My), which has an optimum *T*_G_ = 10 °C and *P*_G_ = 800 bar [42] but is found at depths of 11 km, was found to remain steadily active up to ~1 kbar, which corresponds to the pressure where it was isolated [30] in contrast to MpDHFR, which begins to lose activity at a much lower pressure, ~500 bar, near its *P*_u_ = ~700 bar [27]. Notably, there are only four sequence differences between MpDHFR and MyDHFR. While the absolute activity and catalytic efficiency [27,30] of MyDHFR are greater than that of MpDHFR, how MyDHFR can maintain activity at pressures far beyond the unfolding pressure of MpDHFR is important in understanding pressure adaptations.

All-atom MD simulations at 279 K and 1 and 800 bar of dihydrofolate reductase bound by the cofactor NADPH and substrate DHF, which is the presumed Michaelis complex [21], from the moderate piezophile *M. profunda* and hyperpiezophile *M. yayanosii* were performed to explore adaptations for high pressure, focusing on sequence differences between these homologous enzymes. Note that a consensus sequence numbering of aligned *Moritella* DHFR sequences with *E. coli* DHFR is used in this text (Figure S1), with the original *Moritella* DHFR sequence numbering in parentheses for reference. Of the four sequence differences between the two, the focus is on the residues that are unique to MyDHFR in comparison to all of the *Moritella* DHFR [41]; specifically, the unique residues of MyDHFR, which are Tyr103 (105), Ile119 (121), and His132 (134), while MpDHFR has Cys103, Thr119, and Asn132. General properties such as average mean-square fluctuations, radius of gyration, and hydrogen bonds, as well as QHA, were used to compare compressibilities. In addition, a cavity analysis was used to compare differences in cavity behavior near the unique residues of MyDHFR.

## 2. Materials and Methods

Methods have been described previously [41], so they are described briefly here and in more detail in Supplemental Material. Coordinate manipulations and analyses were performed using the molecular mechanics package CHARMM [43]. Because of the large amount of literature on *E. coli* DHFR, consensus sequence numbering based on the sequence numbering of *E. coli* DHFR is used (Figure S1). Residues 1 and 67 of the original MpDHFR sequence were renumbered to 0 and 66.5, respectively, to be consistent with gaps in the alignment with *E. coli* DHFR. Coordinates of MpDHFR bound by NADP^+^ and folate (PDB: 2ZZA), a presumed analog of the Michaelis complex, were obtained from the PDB [27]. The first residue of the structure was incorrectly determined to be Val, so the first residue was corrected to Met, and the C-terminal tail was built (K160), using GalaxyFill [44] in PDB Reader. For MyDHFR, mutations to the MpDHFR template structure (C103Y, T119I, N132H, N150D) were also made using GalaxyFill. Coordinates of NADP^+^ and folate were modified to the Michaelis complex cofactor NADPH and substrate DHF, respectively, using Ligand Reader and Modeler [45] in CHARMM-GUI. The CHARMM36 all-atom non-polarizable potential energy parameter set was used to model the protein [46,47], water was modeled by TIP4P-Ew [48], the ligand DHF was modeled by CHARMM General Force Field (CGenFF) [49], and cofactor NADPH was modeled by the nucleotide parameter set [50]. MD simulations of *M. profunda* and *M. yayanosii* DHFR at *T* = 279 K and *P* = 1, 800 bar were performed using the molecular mechanics package OpenMM [51]. Each system was minimized with 500 iterations of the L-BFGS algorithm [52]. Heating, pressurization, and an initial 5 ns equilibration were performed in the *NPT* ensemble using a leapfrog Verlet integrator with a time step of 0.001 ps, Andersen thermostat [53] and Monte Carlo (MC) barostat [54]. Afterward, a second 5 ns equilibration followed by 50 ns production was performed in the *NVT* ensemble using a velocity Verlet integrator with a timestep of 0.001 ps and a Nosé-Hoover thermostat [55,56,57,58].

Average properties were calculated from coordinates written at 1 ps intervals except as noted. Averages and standard deviations were calculated by block averaging over 10 ns blocks. The mean-squared fluctuations of the protein-heavy atoms 〈Δ*r*_HA_^2^〉 were calculated within 10 ns blocks with respect to the average structure within each block and then averaged over all blocks. The mean-squared fluctuations of C_α_ atoms, 〈Δ*r*_Cα_^2^〉, were calculated from the entire 50 ns production run with respect to the average structure over the entire production run.

Hydrogen bonds were defined as having a distance between the donor hydrogen atom and acceptor atom smaller than 2.40 Å [59] and an angle of D–H…A larger than 130°. Chemically equivalent donors or acceptors of the same residue were combined, and bifurcated hydrogen bonds were treated as a single event. Further details on hydrogen bond calculations can be found in Supplemental Material.

Cavities and clefts were calculated using defaults, except, as noted, using McVol [60]. A search grid of 1.0 Å, probe radius of 1.1 Å and a refinement grid of 0.5 Å with 50 Monte Carlo steps per Å^3^ were used. Volumes were calculated from structures at every 5 ns from the 50 ns simulation. The average volume and root mean square (RMS) fluctuations in volumes for each cavity or cleft were then obtained from volumes over the ten structures, where volumes less than the 1.0 Å^3^ cutoff were given a volume of 0 Å^3^. Since many cavities transitioned between cavities and solvent-accessible clefts, they were termed as “cavity” or “cleft” based on the larger population.

A quasiharmonic analysis [39,40] from a *P-T* grid of short, 1 ns simulations in the *NPT* ensemble (with coordinates saved every 0.1 ps) starting from the end of the 5 ns *NVT* equilibration at *P* = 1 bar, *T* = 279 K. This *P–T* grid was comprised of all combinations of *P* = 1, 2500, 5000, 7500, and 10,000 bar and *T* = 40, 80, 120, 160, 200, 240, 280, 320 K.

The average fluctuations, *σ*^2^(*P*,*T*), were calculated for each of the 1 ns *P−T* grid simulations. *σ*^2^(*P*,*T*) were calculated by averaging fluctuations with respect to the average structure of each 10 ps interval and then performing a second average over the 100 × 10 ps intervals in the 1 ns simulation. The fits to the quasiharmonic equations were performed in gnuplot. The reference state is *P*_0_ = 1 bar and *T*_0_ = 279 K. First, *σ*^2^(*P*,*T*), from the MD simulation data at all pressures and *T* ≥ 200 K were fit using Equation (1) to *σ*_0_*^2^*, *α_P_*_,0_, and *κ_T_*_,0_.

(1) $$\sigma^{2}\left(P,T\right)=\sigma_{0}{}^{2}\frac{T}{T_{0}}{\left[\left(1+\kappa_{T,0}\Delta P\right)e^{-\alpha_{P,0}\Delta T}\right]}^{-2/3}$$

Next, *σ*^2^(*P*,*T*) from the MD simulation data for all pressures and *T* < 200 K were fit using Equations (2) and (3) to find values for *T*_g,0_ and *c*.

(2) $$\sigma_{g}{}^{2}\left(P\right)=\sigma_{0}{}^{2}\left(\frac{T_{g}\left(P\right)}{T_{0}}\right){\left[\left(1+\kappa_{T,0}\Delta P\right)e^{-\alpha_{P,0}\left(T_{g}\left(P\right)-T_{0}\right)}\right]}^{-2/3}$$

(3) $$T_{g}\left(P\right)=T_{g,0}-c\Delta P$$

## 3. Results

### 3.1. Average Properties

The average properties of the *Moritella* DHFRs in the 50 ns MD simulation are similar and do not change much with pressure (Table 1). At 1 bar, there are three fewer hydrogen bonds in MpDHFR compared to that of MyDHFR, while at 800 bar, there is one more hydrogen bond in MpDHFR than MyDHFR, although error bars are large. The radius of gyration, *R_g_*_,_ for MyDHFR appears slightly larger than for MpDHFR (Table 1).

**Figure 1:**
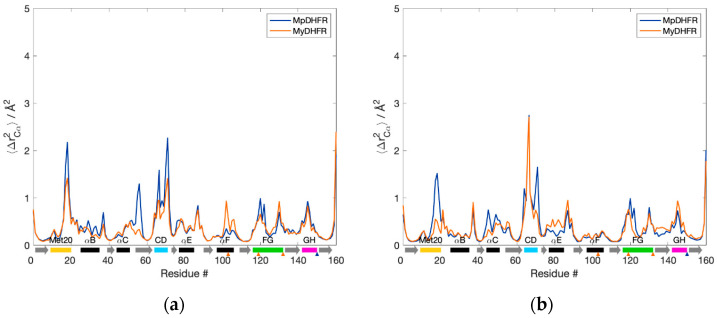
Backbone fluctuations of *Moritella* DHFRs for the 50 ns simulation at (**a**) 1 and (**b**) 800 bar. α-helices (black rectangles), β-strands (gray arrows), and loops (colored rectangles) are denoted below the axis. Unique residues for MpDHFR (blue) and MyDHFR (orange) are noted with triangles below the axis.

The C_α_ fluctuations, 〈Δ*r*_Cα_^2^〉, as a function of residue number are also quite similar (Figure 1). At 1 bar, MpDHFR appears to have slightly higher fluctuations in the Met20, CD, FG loops, and helix C, while MyDHFR has larger fluctuations in helix F (Figure 1a). At increasing pressure, these fluctuations appear to remain unchanged or decrease for MpDHFR, while fluctuations in the CD loop, helix E, and strand G increase for MyDHFR (Figure 1b).

### 3.2. Hydrogen Bonding

**Figure 2:**
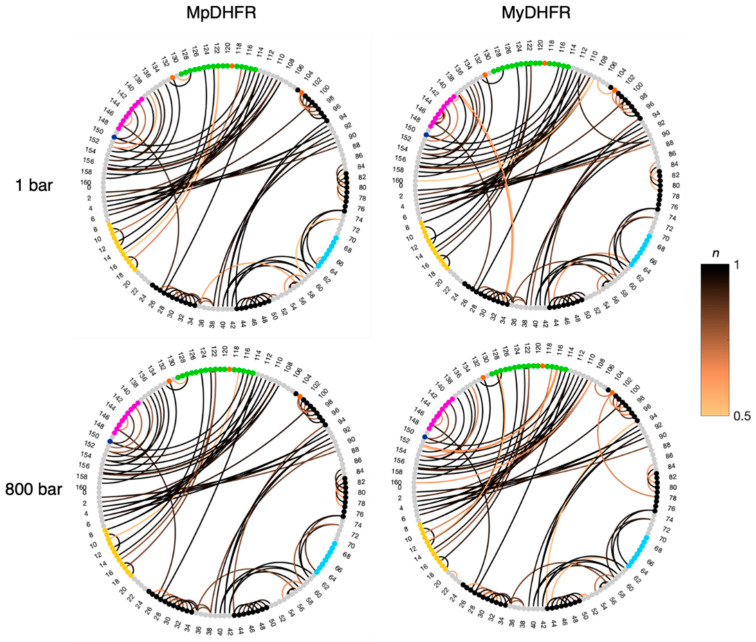
Hydrogen bond occupancy (*n*) for *Moritella* DHFRs at low and high pressure. Connections between donor/acceptor residues with *n* > 0.5 are shown with the line color proportional to the occupancy of the hydrogen bond. The unique residues for Mp- (dark blue) and MyDHFRs (orange) along with the α-helices (black), and Met20 (yellow), CD (cyan), FG (green), and GH (magenta) loops are identified on the nodes.

As a whole, the hydrogen bonding in MpDHFR and MyDHFR at 1 bar is also quite similar (Figure 2), as to be expected given the high degree of homology. However, a prominent difference between Mp- and MyDHFRs involves Res103 (105), where the intrahelical Cys103 (105) S_γ_…Ile99 (101) O hydrogen bond within helix F in MpDHFR is replaced by the Tyr103 (105) O_η_…Leu78 (80) O hydrogen bond between helix F and E in MyDHFR, especially at 800 bar (Table S2).

### 3.3. Potential Energy Landscape

The QHA indicates that the potential energy landscapes for MyDHFR and MpDHFR at 279 K and 1 bar are also quite similar (Table 2). However, the wells are somewhat shallower (*σ*_0_^2^ is greater) in MyDHFR than MpDHFR. Interestingly, the thermal property *α_P_*_,0_ is nearly identical between the two *Moritella* DHFRs, while the compressibility *κ_T_*_,0_ is slightly greater for MyDHFR than MpDHFR, and barriers between wells *T*_g,0_ are somewhat lower for MyDHFR than MpDHFR (Table 2).

### 3.4. Cavities and Clefts

**Figure 3:**
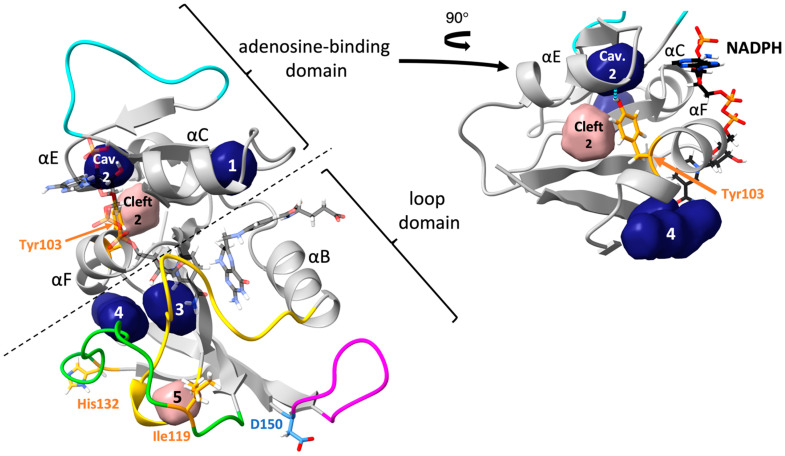
Cavities (dark blue) and clefts (pink) in *Moritella* DHFRs. Ribbon representation of MyDHFR. Unique residues of MyDHFR shown in wire. Cavities discussed in text near-unique residues of MyDHFR identified by arrows. Ligands NADPH and DHF shown in transparent CPK. Met20 (yellow), CD (cyan), FG (green) and GH (magenta) loops are also indicated. Right inset: rotated depiction of adenosine-binding domain with Tyr103 O*_η_*…Leu78 O hydrogen bond (blue dashed line).

**Figure 4:**
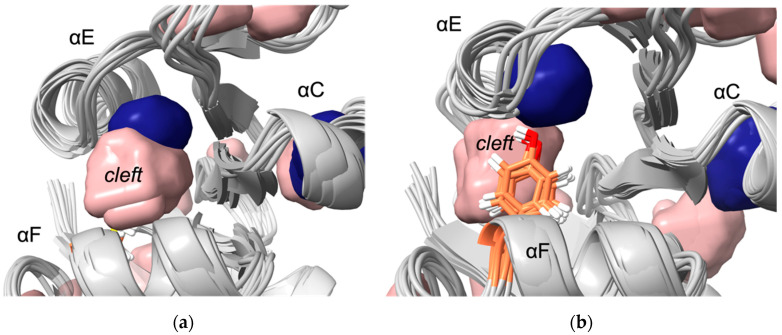
Cavity 2 and cleft 2 near Res103. (**a**) Cavity 2 (dark blue) and solvent-accessible cleft 2 (pink) along helix E across from Cys103 in MpDHFR merge at 800 bar, providing solvent access to the internal cavity. (**b**) Cleft 2 remains separated from cavity 2 by Tyr103 (orange) in MyDHFR, possibly preventing solvent penetration into the cavity.

Five cavities or clefts were found near the unique residues of MyDHFR (Figure 3). The average and root mean square (RMS) fluctuations in the volumes are given in Table 3. Note that the RMS fluctuations are not the error in the average volume but rather the range in values to expect since the volumes are a fluctuating quantity in the simulation. For reference, cavity 1 is found consistently for both DHFR at both pressures. Cavity 1 appears to be relatively independent of species or pressure, with relatively small fluctuations in cavity size. Cleft 2 is found near the unique residue Tyr103 (105) of MyDHFR. Additionally, cavity 2, which can become as large as 9 to 17 Å, appears adjacent to cleft 2, although as a rare event seen in one or at most two of the snapshots and not in MyDHFR at 1 atm. In MpDHFR, both cleft 2 and cavity 2 appear as separate cavities, although they can occasionally merge into a single pathway to the surface (Figure 4) and is also observed in the starting crystal structure (Table S2). However, in MyDHFR, Tyr103 of helix F appears to separate cleft 2 from cavity 2 for much of the simulation while it is hydrogen-bonded to Leu78 (80) of helix E. The Tyr103 ring fills much of the space in which cleft 2 in MpDHFR occupies, shifting and separating cleft 2 from cavity 2. This hydrogen bond becomes stronger at the higher pressure, from an occupancy of ~0.3 at 1 bar to ~0.7 at 800 bar. Furthermore, an inspection of simulations of MyDHFR at 1 bar indicates that when the Tyr103 O*_η_*…Leu78 O hydrogen bond breaks, Tyr103 flips toward the surface of the protein away from helix E, allowing helix E to slip downwards toward the conformation adopted by MpDHFR.

Cavities 3 and 4 are located near the unique residue 132 (134) of MyDHFR, which is at the end of the FG loop, and cleft 5 is embedded within the Met20 and FG loops, adjacent to the unique residue 119 (121) of MyDHFR. Average volumes of cavities 3, 4, and 5 are similar for both proteins and appear to be largely pressure-independent. While cavities 3 and 4 are not observed in the crystal and starting structures (Table S2), this may be accounted for by differences in sidechain conformations due to cryocooling [61] and enhanced solution-state motions at higher temperatures [62]. Crystal structures obtained at various pressures of ternary Michaelis-analog *E. coli* DHFR observed all cavities identified here [63]. MpDHFR has a hydrophilic threonine for Res119, whereas MyDHFR has hydrophobic isoleucine. Inspection of the crystal structure shows a resolved water molecule within cleft 5, while none were resolved within other cavities and clefts.

## 4. Discussion

Pressure leads to a decreased total volume of the system, which can occur by decreasing the total cavity volume or increasing the hydration of cavities. QHA indicates that the potential energy landscape of MyDHFR may be slightly more compressible than MpDHFR due to slightly shallower potential energy wells (indicated by greater *σ*_0_^2^) and lower barriers between wells (indicated by lower *T*_g,0_) as well as slightly larger *R_g_*. However, the difference in the intrinsic isothermal compressibility between the two of 5 × 10^−3^/kbar is much less than between EcDHFR and MpDHFR of 9 × 10^−3^/kbar. Note that the previous work comparing compressibilities [40] is for binary THF-bound DHFR while the current work is of ternary NADPH- and DHF-bound DHFR, so the magnitudes are somewhat smaller here, which is expected as there are more bound ligands and cofactors, but not enough to account for the larger differences between EcDHFR and MpDHFR than between MpDHFR and MyDHFR. Thus, while both MpDHFR and MyDHFR are both expected to have larger compressibilities compared to EcDHFR, this is consistent with the idea that this is mainly an adaptation for the cold that is fortuitously favorable for high pressure. However, the compressibility of MpDHFR may be reaching the limit of increased compressibility possible without destabilizing too much for deeper pressures.

Interestingly, the three unique residues of MyDHFR were found near cavities or clefts. Note that the most important factor here is not the average volumes but the fluctuations in the volumes. For instance, while cavity 2 and cleft 2 are generally separate in MpDHFR, occasionally, the two connect and form a pathway from the surface to the interior. In other words, occasional fluctuations could allow water to penetrate the interior, which could lead to distortion of the structure. However, in MyDHFR, Tyr103 of helix F forms a hydrogen bond with Leu78, which separates the two cavities, thus blocking the pathway, especially at the higher pressure, while in MpDHFR, Cys103 forms an intrahelical hydrogen bond so that the open pathway remains accessible to surrounding solvent.

## 5. Conclusions

Potential adaptations of piezophile proteins appear to have opposite means of being accomplished: greater compressibility by larger total cavity volume versus greater stability against pressure denaturation by smaller cavities. Here, MpDHFR and MyDHFR appear to have similarly large compressibilities, which may be an adaptation for cold (i.e., greater compressibility is also associated with greater flexibility) that fortuitously also makes them function better at high pressure. However, while greater flexibility/compressibility is generally accomplished by fewer or weaker interactions, there may be a limit to how much the flexibility/compressibility can be increased while maintaining the three-dimensional fold so that these two DHFRs may have reached that limit. Moreover, since MpDHFR begins to show deactivation at ~500 bar and unfolding at ~700 bar, the higher pressures that MyDHFR experiences are more likely to result in water penetration. Thus, the evolutionary driver may be preventing water penetration rather than increasing compressibility. Since cleft 2 and cavity 2 can form a pathway from the surface to the interior in MpDHFR, the unique Tyr103 (105) in MyDHFR, which forms a hydrogen bond that prevents the merging of cleft 2 and cavity 2, may be an adaptation to block water from reaching the interior by this pathway.

## Figures and Tables

**Table 1:** Average properties from the 50 ns MD simulation. Average heavy atom mean-square fluctuations, 〈Δ*r*_HA_^2^〉, number of hydrogen bonds, *N*_HB_, and radii of gyration, *R*_g_.

Protein	*P* (bar)	〈Δ*r*_HA_^2^〉 (Å^2^)	*N*_HB_	〈*R*_g_〉 (Å^2^)
MpDHFR	1	0.60 ± 0.04	104 ± 1	15.48 ± 0.07
MpDHFR	800	0.61 ± 0.02	106 ± 3	15.50 ± 0.04
MyDHFR	1	0.60 ± 0.06	107 ± 1	15.63 ± 0.03
MyDHFR	800	0.61 ± 0.08	105 ± 1	15.56 ± 0.05

**Table 2:** Parameters calculated from QHA. Goodness-of-fit given by reduced *χ*^2^.

DHFR	*σ*_0_^2^ (Å^2^)	*𝛼_P_*_,0_ (10^−3^/K)	*𝜅_T_*_,0_ (10^−3^/kbar)	𝜒^2^ (10^−6^)	*T*_g,0_ (K)	–*c* (K/kbar)	𝜒^2^ (10^−6^)
MpDHFR	0.148 ± 0.001	8.0 ± 0.1	65 ± 2	3.5	191 ± 2	0.5 ± 0.3	0.51
MyDHFR	0.151 ± 0.001	8.1 ± 0.1	70 ± 3	4.0	186 ± 1	1.0 ± 0.2	0.39

**Table 3:** Average and RMS fluctuations cavity or cleft volumes, *V*_cav_ (Å^3^).

DHFR	*P* (bar)	Cavity/Cleft					
		1	Cavity 2	Cleft 2	3	4	5
MpDHFR	1	17 ± 8	1 ± 3	5 ± 7	19 ± 5	12 ± 9	4 ± 5
MpDHFR	800	15 ± 5	1 ± 0	3 ± 8	19 ± 3	11 ± 6	2 ± 3
MyDHFR	1	16 ± 6	0 ± 0	10 ± 13	19 ± 5	10 ± 12	8 ± 5
MyDHFR	800	16 ± 3	2 ± 5	8 ± 8	19 ± 5	15 ± 17	2 ± 3

## Data Availability

The data that support the findings of this study are available from the corresponding author upon reasonable request.

## Supplementary Materials

The following are available online at www.mdpi.com/article/10.3390/biology10111211/s1: Detailed methods section, Figure S1: Sequence alignment of MpDHFR and MyDHFR with consensus sequence numbering, Table S1: Starting system information, Table S2: Cavities and clefts for crystal and starting structures, and Table S3: Differences in hydrogen bonding involving residue 103.
