# Supplementary material for: Pressure Adaptations in Deep-Sea Moritella Dihydrofolate Reductases: Compressibility versus Stability
Source: Biology (Basel). 2021 Nov 20;10(11):1211. doi: 10.3390/biology10111211 (PMC8614765; doi:10.3390/biology10111211)
Supplement: Supplementary file 1 [file biology-10-01211-s001.zip › biology-1450723-supplementary.pdf]

Pressure Adaptations in Deep-sea *Moritella* Dihydrofolate Reductases: Compressibility versus Stability

Ryan Penhallurick and Toshiko Ichiye

SUPPLEMENTAL MATERIAL

S1. Methods

Coordinate manipulations and analyses were performed using the molecular mechanics package CHARMM version 40b1 [1]. Molecular dynamics (MD) simulations were performed using the molecular mechanics package OpenMM version 7.3.1 [2] compiled with CUDA version 9.2. The CHARMM36 all-atom non-polarizable potential energy parameter set was used to model the protein [3,4]. Water was modeled by TIP4P-Ew because of the importance of modeling changes in the properties of water under pressure [5]. A CHARMM General Force Field (CGenFF) was generated for DHF through ParamChem (v. 1.0.0) [6] with hydrogen bonding lists added manually. The force field developed by Mackerell *et. al.* [7] was used to describe the reduced cofactor NADPH. Sequences were aligned using ClustalX v.2.1 [8]. Ligand Reader and Modeler [9] in CHARMM-GUI [10] was used to modify the pterin ring of folate from a planar system to the partially-puckered ring of dihydrofolate (DHF), as well as to modify oxidized nicotinamide adenine dinucleotide phosphate ($NADP^+$) to the reduced form (NADPH).

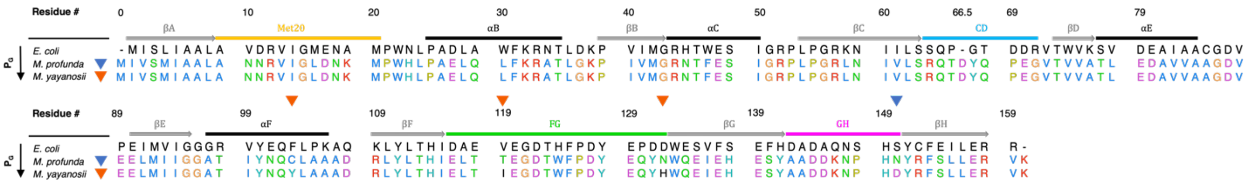

Figure S1. Consensus sequence alignment for MpDHFR and MyDHFR. Unique residues are indicated by triangles for MpDHFR (blue) and MyDHFR (orange). Secondary structure elements are indicated by horizontal bars: α-helices (black), β-strands (gray arrows) and the Met20 (yellow), CD (blue), FG (green) and GH (magenta) loops. Sequence numbering is based on *E. coli* DHFR (black sequence), with the first residue denoted as 0 and the gap between residues 65 and 66 of *E. coli* DHFR denoted as 66.5.

Coordinates for the proteins were generated with PDB Reader [11]; specifically, termini were capped with amino and carboxyl groups, and missing hydrogen coordinates built. Coordinates of MpDHFR bound to $NADP^+$ and folate (PDB: 2ZZA), were obtained from the PDB [12]. Sequence numbering is based on alignment with *E. coli* DHFR (Figure S1). Residues 1 and 67 of the original MpDHFR sequence were renumbered to 0 and 66.5, respectively, to be consistent with gaps in the alignment with *E. coli* DHFR. The first residue of the structure was incorrectly determined to be Val, so the first residue was corrected to Met, and the C-terminal tail was built (K160), using GalaxyFill [13] in PDB Reader. For MyDHFR, mutations to the MpDHFR template structure (C103Y, T119I, N132H, N150D) were also made using GalaxyFill. Coordinates of $NADP^+$ and ligand folate were modified to the Michaelis cofactor NADPH and substrate DHF, respectively, using Ligand Reader and Modeler [9] in CHARMM-GUI. Crystal waters within 2.5 Å of any modeled residue were deleted. The DHFRs were solvated in a cubic simulation box of equilibrated TIP4P-Ew with a distance between faces of ~70 Å. Solvent waters within 2.5 Å of any crystal water, ligand or protein heavy atom were deleted. The smallest distance from a protein atom to a side of the box was ~10

Å. The proteins were then neutralized in 0.15 M KCl using the Monte-Carlo placement method. System details can be found in Table S1.

The subsequent calculations were performed in OpenMM as described briefly here; in particular, changes from default settings are noted. The calculations were "mixed precision," in which forces and integration are calculated in single and double precision, respectively. Nonbonded interactions had a cutoff of 12 Å, with the Leonard-Jones interactions switched off smoothly using the default OpenMM switching function beginning at 10 Å to the cutoff and no long-range corrections. The particle mesh Ewald (PME) summation algorithm [14], with an Ewald error tolerance of 1×10^{-5}, was used for the electrostatics. Each system was minimized with a harmonic restraint with a force constant of 100 kcal mol^{-1} $Å^{-2}$ for 500 iterations of the L-BFGS algorithm [15]. Initial stages of the simulations were performed using a leapfrog Verlet integrator with a time step of 0.001 ps and were maintained in the *NPT* ensemble using an Andersen thermostat [16] updated every 1000 steps and Monte Carlo (MC) barostat [17] updated every 25 steps. Each system was heated from an initial temperature of 0 K to the final temperature in 5 K intervals of 5 ps each, followed by pressurization from 1 bar to the final pressure in 20 bar intervals of 20 ps each. A harmonic restraint with a force constant of 5 kcal mol^{-1} $Å^{-2}$ was applied to the heavy atoms of the protein and ligands during heating and pressurization [18], and then gradually decreased from 5 to 0 kcal mol^{-1} $Å^{-2}$ in 0.5 kcal mol^{-1} $Å^{-2}$ intervals for a total of 20 ps. Next, the system was equilibrated for 5 ns in the *NPT* ensemble with all harmonic restraints removed. The final stages of the simulations were performed utilizing a velocity Verlet integrator with a timestep of 0.001 ps maintained in the *NVT* ensemble using a Nosé-Hoover thermostat [19-22]. All simulations were run for an additional 100 ps and the system volumes every 1 ps were compared to that of the average volume from the last 4 ns of the *NPT* equilibration run. For all simulations at 1 bar, the closest volume less than the average volume of the *NPT* equilibration run was used to start the *NVT* production run; while for all other conditions the closest volume to the average of the *NPT* run was chosen. The system was equilibrated for another 5 ns followed by 50 ns of production run in the *NVT* ensemble.

Table S1. Starting information for simulations. Unique residues identified from sequence alignement of *Moritella* DHFRs [23]. Total number of atoms within the system, $N_{atoms,tot}$, and protein, $N_{atoms,prot}$. Total waters, N_{wat}, potassium ions, N_{K+} and chloride ions N_{Cl-}.

Protein	Unique Residues	$N_{atoms,tot}$	$N_{atoms,prot}$	N_{wat}	N_{K+}	N_{Cl-}
MpDHFR	D150N	43515	2558	10191	40	27
MyDHFR	C103Y, T119I, N132H	43456	2574	10172	41	27

Hydrogen bonds were defined as having a distance between the donor hydrogen atom *i* and acceptor atom *j* smaller than 2.40 Å [24] and an angle of D–H···A larger than 130°. The time-averaged number of hydrogen bonds N_{HB} was calculated as the average number of hydrogen bonds at each timestep. Hydrogen bonding events were calculated in CHARMM, while MATLAB was used to calculate the average occupancies and lifetimes for each hydrogen bond pair. Two hydrogen bonds simultaneously formed with the same protein atom were calculated as two separate events. For chemically equivalent hydrogen bonding donors or acceptors of the same residue, equivalent atoms (such as $O_{\delta1}$/$O_{\delta2}$ in Asp) were combined. The occupancy n_{ij} was defined as the fraction of the total simulation time in which *i* and *j* are hydrogen bonded. Bifurcated hydrogen bonds were treated as a single event so that the maximum occupancy would be one. The average hydrogen bond lifetime, τ_{ij}, is the sum of the time, t_{ij}, that donor atom *i* is in a hydrogen bond with any acceptor atom *j*, over the number of hydrogen bonding events, n_{ij},

$$\tau_{ij} = \frac{1}{n_{ij}} \sum_{i=1}^{n_{ij}} t_{ij}(n) \qquad \text{(S1)}$$

The average overall hydrogen bond lifetime between species α and β for a simulation, $\tau_{\alpha\beta}$, is

$$\tau_{\alpha\beta} = \frac{1}{N}\sum_{i,j}\tau_{ij} \quad \text{(S2)}$$

where τ_{ij} is the average hydrogen bond lifetime between any atom pair *ij*, respectively, and *N* is the total number of individual hydrogen bond pairs between the two species.

S2. Results

Table S2. Average cavity or cleft volumes, V_{cav} (Å^3) for crystal structure (PDB: 2ZZA [12]**) and starting coordinates for each protein.**

DHFR	Coordinate Set	Cavity / Cleft					
		1	Cavity 2	Cleft 2	3	4	5
MpDHFR	Crystal (PDB: 2ZZA)	10	0	34	0	0	3
MpDHFR	Starting Coordinates	12	0	28	0	0	1
MyDHFR	Starting Coordinates	12	0	28	0	0	0

Table S3. Differences in hydrogen bonding involving Res103.

Donor	Acceptor	MpDHFR		MyDHFR	
		1 bar	800 bar	1 bar	800 bar
Res103 N (αF)	Ile99 O (αF)	0.86 (8.7 ps)	0.83 (6.6 ps)	0.67 (4.3 ps)	0.60 (2.9 ps)
Ala107 N (αF)	Res103 O (αF)	0.00 (0.0 ps)	0.00 (0.0 ps)	0.56 (3.0 ps)	0.73 (4.1 ps)
Cys103 S_γ (αF)	Ile99 O (αF)	0.61* (3.7 ps)	0.70* (4.4 ps)	–	–
Tyr103 O_η (αF)	Leu78 O (αE)	–	–	0.27* (2.6 ps)	0.69* (5.3 ps)

* From previous work [23].

References

1. Brooks, B.R.; Brooks, C.L., III; MacKerell, A.D., Jr.; Nilsson, L.; Petrella, R.J.; Roux, B.; Won, Y.; Archontis, G.; Bartels, C.; Boresch, S., et al. CHARMM: The biomolecular simulation program. *J. Comput. Chem.* **2009**, *30*, 1545-1614.

2. Eastman, P.; Swails, J.; Chodera, J.D.; McGibbon, R.T.; Zhao, Y.; Beauchamp, K.A.; Wang, L.P.; Simmonett, A.C.; Harrigan, M.P.; Stern, C.D., et al. OpenMM 7: Rapid development of high performance algorithms for molecular dynamics. *PLoS Comput. Biol.* **2017**, *13*, e1005659.
3. MacKerell Jr., A.D.; Bashford, D.; Bellot, M.; Dunbrack Jr., R.L.; Field, M.J.; Fischer, S.; Gao, J.; Guo, H.; Ha, S.; Joseph, D., et al. All-atom empirical potential for molecular modeling and dynamics studies of proteins. *J. Phys. Chem. B* **1998**, *102*, 3586-3616.
4. Best, R.B.; Zhu, X.; Shim, J.; Lopes, P.E.; Mittal, J.; Feig, M.; Mackerell, A.D., Jr. Optimization of the additive CHARMM all-atom protein force field targeting improved sampling of the backbone phi, psi and side-chain chi(1) and chi(2) dihedral angles. *J. Chem. Theory Comput.* **2012**, *8*, 3257-3273.
5. Horn, H.W.; Swope, W.C.; Pitera, J.W.; Madura, J.D.; Dick, T.J.; Hura, G.L.; Head-Gordon, T. Development of an improved four-site water model for biomolecular simulations: TIP4P-Ew. *J. Chem. Phys.* **2004**, *120*, 9665-9678.
6. Vanommeslaeghe, K.; Hatcher, E.; Acharya, C.; Kundu, S.; Zhong, S.; Shim, J.; Darian, E.; Guvench, O.; Lopes, P.; Vorobyov, I., et al. CHARMM general force field: A force field for drug-like molecules compatible with the CHARMM all-atom additive biological force fields. *J. Comput. Chem.* **2010**, *31*, 671-690.
7. Pavelites, J.J.; Gao, J.L.; Bash, P.A.; Mackerell, A.D. A molecular mechanics force field for NAD(+), NADH, and the pyrophosphate groups of nucleotides. *J. Comput. Chem.* **1997**, *18*, 221-239.
8. Larkin, M.A.; Blackshields, G.; Brown, N.P.; Chenna, R.; McGettigan, P.A.; McWilliam, H.; Valentin, F.; Wallace, I.M.; Wilm, A.; Lopez, R., et al. Clustal W and Clustal X version 2.0. *Bioinformatics* **2007**, *23*, 2947-2948.
9. Kim, S.; Lee, J.; Jo, S.; Brooks, C.L.; Lee, H.S.; Im, W. CHARMM-GUI ligand reader and modeler for CHARMM force field generation of small molecules. *J. Comput. Chem.* **2017**, *38*, 1879-1886.
10. Jo, S.; Kim, T.; Iyer, V.G.; Im, W. CHARMM-GUI: A web-based graphical user interface for CHARMM. *J. Comput. Chem.* **2008**, *29*, 1859-1865.
11. Jo, S.W.; Cheng, X.; Islam, S.M.; Huang, L.; Rui, H.; Zhu, A.; Lee, H.S.; Qi, Y.F.; Han, W.; Vanommeslaeghe, K., et al. CHARMM-GUI PDB Manipulator for Advanced Modeling and Simulations of Proteins Containing Nonstandard Residues. In *Biomolecular Modelling and Simulations*, KarabenchevaChristova, T., Ed. Elsevier Academic Press Inc: San Diego, 2014; Vol. 96, pp. 235-265.
12. Ohmae, E.; Murakami, C.; Tate, S.-i.; Gekko, K.; Hata, K.; Akasaka, K.; Kato, C. Pressure dependence of activity and stability of dihydrofolate reductases of the deep-sea bacterium *Moritella profunda* and *Escherichia coli*. *Biochim. Biophys. Acta* **2012**, *1824*, 511-512.
13. Coutsias, E.A.; Seok, C.; Jacobson, M.P.; Dill, K.A. A kinematic view of loop closure. *J. Comput. Chem.* **2004**, *25*, 510-528.

14. York, D.M.; Pedersen, L.G.; Darden, T.A. The effect of long-range electrostatic interactions in simulations of macromolecular crystals: A comparison of the Ewald and trucated list methods. *J. Chem. Phys.* **1993**, *99*, 8345-8348.
15. Liu, D.C.; Nocedal, J. On the Limited Memory BFGS Method for Large-Scale Optimization. *Math. Program.* **1989**, *45*, 503-528.
16. Andersen, H.C. Molecular-dynamics simulations at constant pressure and/or temperature. *J. Chem. Phys.* **1980**, *72*, 2384-2393.
17. Aqvist, J.; Wennerstrom, P.; Nervall, M.; Bjelic, S.; Brandsdal, B.O. Molecular dynamics simulations of water and biomolecules wit a Monte Carlo constant pressure algorithm. *Chem. Phys. Lett.* **2004**, *384*, 288-294.
18. Sheinerman, F.B.; Brooks, C.L. A molecular dynamics simulation study of segment B1 of protein G. *Protein Struct. Funct. Genet.* **1997**, *29*, 193-202.
19. Nosé, S. A unified formulation of the constant temperature molecular dynamics methods. *J. Chem. Phys.* **1984**, *81*, 511-519.
20. Hoover, W.G. Canonical dynamics: Equilibrium phase-space distributions. *Phys. Rev. A* **1985**, *31*, 1695-1697.
21. Martyna, G.J.; Klein, M.L.; Tuckerman, M. Nose-Hoover Chains - The Canonical Ensemble via Continuous Dynamics. *J. Chem. Phys.* **1992**, *97*, 2635-2643.
22. Martyna, G.J.; Tuckerman, M.E.; Tobias, D.J.; Klein, M.L. Explicit reversible integrators for extended systems dynamics. *Mol. Phys.* **1996**, *87*, 1117-1157.
23. Penhallurick, R.W.; Durnal, M.D.; Harold, A.; Ichiye, T. Adaptations for Pressure and Temperature in Dihydrofolate Reductases. *Microorganisms* **2021**, *9*, 1706.
24. de Loof, H.; Nilsson, L.; Rigler, R. Molecular dynamics simulation of galanin in aqueous and nonaqueous solution. *J. Am. Chem. Soc.* **1992**, *114*, 4028-4035.
